# Differential Effects of Maternal High Fat Diet During Pregnancy and Lactation on Taste Preferences in Rats

**DOI:** 10.3390/nu12113553

**Published:** 2020-11-20

**Authors:** Gabor C. Mezei, Serdar H. Ural, Andras Hajnal

**Affiliations:** 1 MFM Fellowship Training Program, Division of Maternal Fetal Medicine, Department of Obstetrics & Gynecology, The Pennsylvania State University College of Medicine, Hershey, PA 17033, USA; mezgab@yahoo.com (G.C.M.); sural@pennstatehealth.psu.edu (S.H.U.); 2 Department of Neural and Behavioral Sciences, The Pennsylvania State University College of Medicine, Hershey, PA 17033, USA

**Keywords:** high fat diet, obesity, taste preferences, oral and post-oral feedback, pregnancy, lactation

## Abstract

Maternal intake of high fat diet (HFD) increases risk for obesity and metabolic disorders in offspring. Developmental programming of taste preference is a potential mechanism by which this occurs. Whether maternal HFD during pregnancy, lactation, or both, imposes greater risks for altered taste preferences in adult offspring remains a question, and in turn, was investigated in the present study. Four groups of offspring were generated based on maternal HFD access: (1) HFD during pregnancy and lactation (HFD); (2) HFD during pregnancy (HFD-pregnancy); (3) HFD during lactation (HFD-lactation); and (4) normal diet (ND) during pregnancy and lactation (ND). Adult offspring 70 days of age underwent sensory and motivational taste preference testing with various concentrations of sucrose and Intralipid solutions using brief-access automated gustometers (Davis-rigs) and 24 h two-bottle choice tests, respectively. To control for post-gestational diet effects, offspring in all experimental groups were weaned on ND, and did not differ in body weight or glucose tolerance at the time of testing. Offspring exposed to maternal HFD showed increased sensory taste responses for 0.3, 0.6, 1.2 M sucrose solutions in HFD and 0.6 M in HFD-pregnancy groups, compared to animals exposed to ND. Similar effects were noted for lower concentrations of Intralipid in HFD (0.05, 0.10%) and HFD-pregnancy (0.05, 0.10, 0.5%) groups. The HFD-lactation group showed an opposite, diminished responsiveness for sucrose at the highest concentrations (0.9, 1.2, 1.5 M), but not for Intralipid, compared to ND animals. Extended-access two-bottle tests did not reveal major difference across the groups. Our study shows that maternal HFD during pregnancy and lactation has markedly different effects on preferences for palatable sweet and fatty solutions in adult offspring and suggests that such developmental programing may primarily affect gustatory mechanisms. Future studies are warranted for determining the impact of taste changes on development of obesity and metabolic disorders in a “real” food environment with food choices available, as well as to identify specific underlying mechanisms.

## 1. Introduction

Obesity is a major risk factor for chronic health conditions and the number one cause of preventable deaths in the US [1]. Despite the large amount of research targeting all the factors which are thought to be involved in the peripheral and central energy balance regulation, the etiology and pathomechanism of obesity is still not well understood.

Obesity appears to be a transgenerational phenomenon driven by both genetic and environmental factors. In the last two decades, interest turned to the early developmental period after Barker et al. introduced the term developmental (fetal) programming [2]. Since then, a large number of human and animal studies have shown that a wide range of sub-optimal perinatal (pregnancy and lactation) nutrition regimens (e.g., high fat, high sugar, low calorie, low protein, low iron, etc.) program basically the same general phenotypic outcomes including obesity and metabolic diseases in adulthood [3,4,5,6].

Our knowledge is limited regarding the mechanisms linking maternal nutrition and developmental changes. Placental transport of essential macronutrients (glucose, amino acids, fatty acids), which is regulated by numerous factors including concentration gradients, placental metabolism, blood flow, and inflammation plays an essential role and has been the target of investigations in both animals [7,8] and humans [9,10]. The early postnatal period during lactation has also been investigated, indicating that maternal high fat diet (HFD) decreases milk production and impairs milk composition [11]. A recent study demonstrated that maternal HFD-induced hypertension in adult offspring may be related to alteration of gut microbiota [12]. It has been also shown that maternal HFD alters sweet taste receptors’ expression in taste buds in rat offspring [13]. Epigenetic alterations (i.e., histone modification and DNA methylation) are thought to be responsible for this by causing an irreversible alteration of the central nervous regulatory centers at the time of peak neural plasticity during early development [14]. Previous studies investigating this developmental effect mainly focused on the hypothalamic centers involved in energy homeostasis regulation [15]. It has been shown that HFD during pregnancy and lactation can increase neurogenesis [16] and permanently alter expression of neuropeptides and receptors [17] in the hypothalamus. However, obesity may also be driven by altered individual taste preferences. Increased taste preferences for highly palatable foods (sugar, salt, fat) can directly lead to stimulatory overeating [18,19], whereas, decreased taste preferences may also lead to overeating although through different compensatory, pleasure seeking behavioral mechanisms [20]. Thus, alteration in taste preferences may be another potential mechanism by which developmental programming of obesity and related metabolic disorders occur. Until now, only a small number of studies were investigating this potential mechanism and able to show an association between perinatal maternal diet and offspring propensity for similar palatable food in animals [21,22] and humans [23,24]. The hedonic aspect of feeding, including sensory taste preference, is thought to be primarily regulated by the midbrain reward-related centers [25,26]. Maternal HFD consumption has been shown to increase palatable food intake and suppress dopamine signaling in offspring [27]. One study showed clear evidence for methylation and gene expression changes in the midbrain dopamine system of the offspring due to maternal HFD exposure in mice [28].

The goal of this study was to investigate the effect of maternal HFD on sensory and motivational taste functions in offspring utilizing brief-access licking tests and long-access two-bottle choice tests, respectively. In addition, by recognizing the potential determining effect of suckling on early development of individual taste preferences, we also focused our investigation to distinguish between gestation and lactation effects. We hypothesized that maternal HFD during pregnancy and lactation differentially alter the sensory taste responses for palatable solutions in adult rat offspring by programming gustatory processes during early development, independent of post-natal diet experience.

## 2. Materials and Methods

### 2.1. Animals

Young female, Sprague Dawley rats from Charles River Laboratories (Horsham, PA, USA) were used in our experiments. Animals were group housed (3/group) in mesh floored, stainless steel hanging cages in a temperature-controlled vivarium while maintained on a constant 12:12 h light-dark cycle (lights on at 0700). Prior to breeding and feeding group assignments, animals were maintained on pelleted normal rat chow *ad libitum*. Filtered tap water also was available ad libitum throughout the experiments. All animal procedures were approved by the Pennsylvania State University College of Medicine Institutional Animal Care and Use Committee and conformed to National Institutes of Health guidelines for the care and use of Laboratory animals (publication No. 8023, revised 1978).

### 2.2. Diets

Two types of diet were used during the experiment. The normal laboratory rat chow (Rodent Diet-W 2018, Harlan Teklad, Madison, WI, USA), referred to as normal diet (ND), has 3.4 kcal/g of energy of which 17% is from fat, 60% from carbohydrates, and 23% is from protein. The high fat diet (HFD) (Research Diet D12492, Research Diets Inc., New Brunswick, NJ, USA) has 5.24 kcal/g of energy with 60% from fat, 20% from carbohydrates, 20% from protein. Animals were maintained on their respective diet ad libitum.

### 2.3. Experimental Design

**Figure 1:**
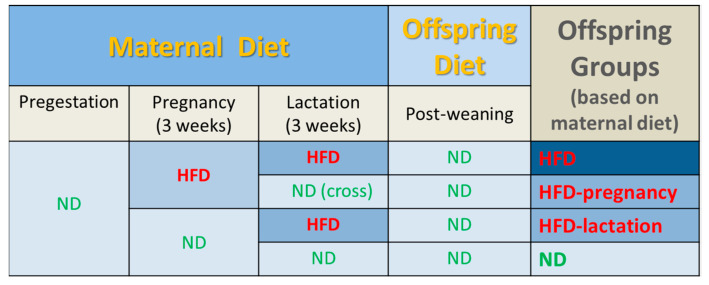
Experimental groups and feeding protocols. Dams were fed either standard rodent chow (normal diet, ND) or high fat diet (HFD) ad libitum during pregnancy from embryonic day 1; *n* = 6/group. At the time of birth, half of the mothers from both groups were switched to the opposite respective diet, generating 4 groups of offspring based on gestational (intrauterine) and lactational (early postnatal) maternal HFD or ND exposure as follows: (1) HFD during gestation and lactation (HFD); (2) HFD during pregnancy and ND during lactation (HFD-pregnancy), with the offspring cross-fostered at birth to ND dams never previously exposed to HFD; (3) ND during pregnancy and HFD during lactation (HFD-lactation); and (4) ND during both gestation and lactation (ND). Following weaning, all offspring were maintained exclusively on ND throughout the course of experiment. Taste testing commenced on postnatal day 70. Abbreviations: HFD, high fat diet; ND, normal diet.

Twelve young, approximately 14 weeks of age (236–275 g) female rats were mated with sexually experienced males (3:1 ratio) and immediately half of the dams (*n* = 6) were placed on HFD for the duration of pregnancy, whereas the other half were kept on ND (*n* = 6). All dams were pregnant, and were then housed individually in solid floored cages. Rats gave birth on days 21–22 of pregnancy, with no differences in the litter size (average of 13 [10,11,12,13,14,15,16] pups per litter), and balanced distribution of sex between the dams fed on HFD or ND during pregnancy. At the time of birth, half of the mothers from both groups were switched to the opposite diet respectively, generating 4 groups of dam (*n* = 3) and ultimately 4 groups of offspring on the basis of gestational (intrauterine) and lactational (early postnatal) exposure to maternal HFD or ND, as follows: (1) HFD during gestation and lactation (HFD); (2) HFD during pregnancy and ND during lactation (HFD-pregnancy); (3) ND during pregnancy and HFD during lactation (HFD-lactation); and (4) ND during both gestation and lactation (ND) (Figure 1). To eliminate the potentially confounding effect of gestational HFD-induced long-term maternal physiological changes, which may encroach on the lactational period and affect fetal development via altered milk composition, offspring of the HFD-pregnancy dams cross-fostered on postnatal day 1 for the period of lactation to ND dams naive to HFD. Cross-fostered pups (*n* = 13) were permanently marked with subcutaneous dye on the base of their tail for easy identification. The pups in the other three groups (HFD, HFD-lactation, and ND) were suckled by their own dams. After birth and cross-fostering, litters were culled to *n* = 8. At the time of weaning (postnatal day 21), three male pups were randomly selected from each litter (*n* = 9/feeding group) for taste experiments. Over the course of the experiment, two pups were excluded from the final analysis due to sickness, both were in the HFD-lactation group, making the final count of pups in this group to be only seven. After weaning, offspring were maintained exclusively on ND throughout their life, including the period of the taste testing experiments, until the time of sacrifice at 14 weeks of age.

### 2.4. Body Weight and Caloric Intake

Maternal body weight was recorded on the day of mating (day 0), on day 7, 14, and 20 during gestation and on day 14 and 21 during lactation. Gestational weight gain was calculated based on the body weight readings at the time of mating (pregestational) and on day 20 of gestation and expressed as percentage of the pregestational weight. Maternal daily caloric intake was calculated once during gestation (pregnancy day 20) and once during lactation (lactation day 16) based on daily food intake measurements (grams) and the total caloric value of the respective diet. Offspring body weight was recorded at birth, at weaning (day 21), and weekly thereafter throughout the entire course of the experiment until litters were sacrificed on day 96 or 97 of life. Offspring daily caloric intake was calculated based on their average daily food intake measurements (grams) during three consecutive days between 68 and 70 days of life, just prior to taste testing.

### 2.5. Intraperitoneal Glucose Tolerance Tests (IPGTT)

IPGTTs were performed on mothers on pregnancy day 14 and on offspring on day 90 and 91 of life, just after taste testing experiments by careful intraperitoneal injections of glucose solution (1 g/kg). The rats were fasted overnight (minimum 16 h) prior the tests. Blood was taken from the tail vein at 0, 15, 30, 60, and 120 min for measurement of blood glucose levels. Blood glucose was determined with a glucometer (Elite Glucometer, Bayer, Elkhart, IN, USA). Animals were classified as diabetic if the peak level of plasma glucose at any time point was 16.8 mmol/L (300 mg/dL) or glucose level at 120 min was greater than 11.2 mmol/L (200 mg/dL).

### 2.6. Taste Assessment Using Brief-Access Tests

Rats were handled daily for a minimum of one week prior to the onset of behavioral experimental procedures. Rats were tested individually by using multi-bottle gustometers (“Davis Rig” from DiLog Instruments, Tallahassee, FL, USA) as previously described [29]. The testing took place during the light phase starting in the morning (~0800) at 10–11 weeks of age. The standard protocol of training and brief-access tests used in our laboratory is described elsewhere [19,30]. Briefly, twenty-four hours before water training, water cylinders were removed from the home cages of all rats. On days 1–5, water training took place; each rat was placed in the gustometer and allowed to lick the drinking spout for water for a 20-min period to familiarize them to the environment and learn to drink from the spout of the gustometer. To maintain proper hydration during water training, all rats received an additional 120 min access period to water in the home cage following each daily session (1500–1700). Following the 5 day water training, on days 6–13, the rats were presented with water and various concentrations of palatable sucrose or Intralipid solutions without overnight water deprivation. First, each rat was tested for sucrose for four consecutive days, followed by testing for Intralipid the same way. In a daily session, six concentrations of sucrose (0.1, 0.3, 0.6, 0.9, 1.2, 1.5 M) or Intralipid (0.05, 0.1, 0.5, 1.0, 5.0, 10.0%) solutions with water were available for 10 s access periods in a randomized order over 20 min. During this period, all concentrations were presented in equal number. A trial was initiated when a rat made a lick within 10 s. The minimum intertrial interval (i.e., between solutions) was 5 s, the amount of time required for the shutter operation and the rig to change positions. The session length was 20 min, given the 80 presentations and the 15 s trials including the intertrial intervals. Data from the first sessions with each stimulus were excluded from the statistical analysis. The rationale for this was to minimize the effect of novelty.

### 2.7. Two-Bottle Choice Preference Tests

Two days following the short access lick-rate tests (12 weeks of age), all four groups of offspring were exposed to two-bottle tests to assess preferences during extended (24 h) exposure for sucrose or Intralipid solutions compared with water. The bottles were alternated (left/right) halfway through the test to prevent a positional bias and light-dark cycle effect. We tested two different concentrations of sucrose (0.3 or 1.0 M) and one concentration of Intralipid (5.0%) solution separately with 1 day off between the tests (i.e., the rats received only water). Two bottles, one filled with tastant (0.3 or 1.0 M sucrose or 5.0% Intralipid) solution and another with water, were used in each setting. Placement of the bottles with tastant and water was randomized across the tests to avoid place preferences. Tastant + water consumptions were measured at 1, 6, 12, and 24 h time periods in each setting. Preference was expressed as ratio of tastant intake over total fluid intake (i.e., tastant and water). A higher than 0.5 preference score is suggestive of a bias towards the tastant. The preference test was carried out with food (ND) available all the time.

### 2.8. Chemicals

Sucrose was purchased from Fisher Scientific (Fair Lawn, NJ, USA), whereas Intralipid from Baxter Healthcare Corporation (Deerfield, IL, USA). Intralipid is a complex nutritive water-soluble emulsion of soybean oil, egg phospholipids, and glycerin with similar viscosity character to sucrose. It is frequently used in rodent studies as a palatable complex fatty stimulus as well as a component of parenteral nutrition for patients who are unable to get nutrition via an oral diet. Sucrose was dissolved in filtered tap water from a source identical to the maintenance water available to the animals in their home cages. Both sucrose and Intralipid solutions were prepared freshly and presented at room temperature.

### 2.9. Statistical Analysis

Maternal food intake and body weight measurements were analyzed using one-way analysis of variance (ANOVA) separately for gestation and lactation. Comparison of gestational weight gain between dams was conducted using two-sample t-test. Offspring body weight and food intake measurements were analyzed using two-way ANOVA. Data points and area under the curve (AUC) from IPGTT tests for each group was calculated and analyzed by comparing between diet groups at each time using either two-sample t-test (dams) or two-way ANOVAs (offspring). In terms of gustometer data, the licks elicited during each 10 s trial were measured, and the mean number of licks for water and for each concentration of chemical was computed for each rat. These means were then used to calculate the difference score between licks made for a given concentration of chemical and those made for water: lick difference score (chemical x) = licks (chemical x) − licks (water). The rationale for this was to control for differential water licks between diet conditions. Two-way ANOVA (main factors: diet groups and concentrations) was conducted on the lick difference score for each tastant. Data from two-bottle tests were also analyzed using two-way ANOVA.

Following the ANOVA, multiple post-hoc pairwise comparisons were conducted between individual diet groups at each concentration of tastants using Tukey’s test. In all analyses, data were presented as mean ±SEM and statistical significance was set at a *p* value < 0.05. Statistical analysis was conducted using GraphPad Prism software (GraphPad Software, Inc. La Jolla, CA, USA).

## 3. Results

### 3.1. Maternal Characteristics

**Figure 2:**
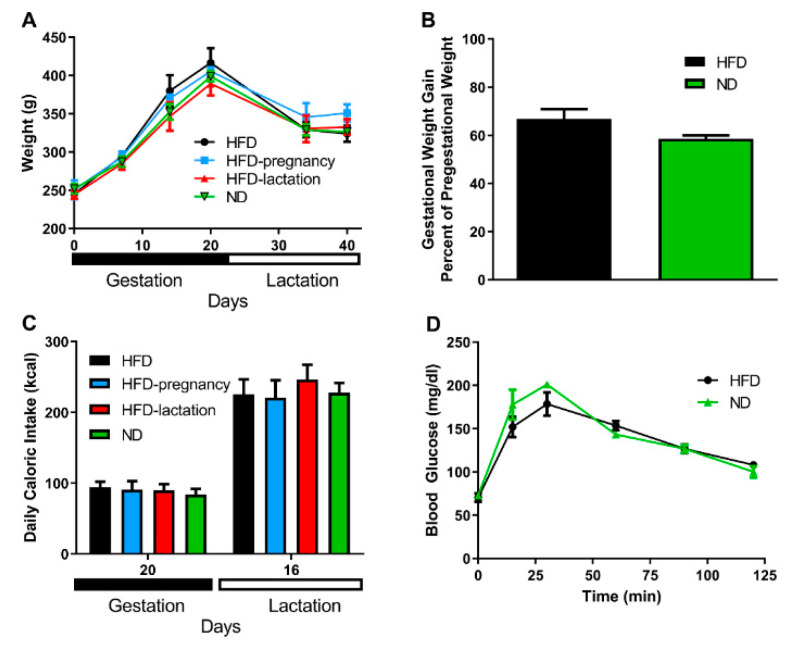
Maternal characteristics. Maternal body weight (**A**). HFD mothers are represented by black symbols and lines; HFD-pregnancy mothers by light blue symbols and lines; HFD-lactation group by red symbols and lines, whereas ND group of mothers were represented by green symbols and lines. Gestational weight gain (**B**). Black columns depict HFD and HFD-pregnancy dams combined; green columns: ND and HFD-lactation dams combined. Maternal daily caloric intake (**C**). Black columns represent HFD dams; light blue columns: HFD-pregnancy dams; red columns: HFD-lactation dams; and green columns: ND group of offspring. Glucose tolerance tests (**D**). Group representations were the same as in graph B, but here with symbols and lines. Data expressed as mean ±SEM. For more details and statistics, see Results.

Maternal body weight was recorded on the day of mating (pregestational weight), on day 7, 14, and 20 of gestation and on day 14 and 21 of lactation (Figure 2A). No significant differences were noted across the groups in any of the examined time points; *n* = 3/group. Gestational weight gain was expressed as percentage of the pregestational weight of the mothers, there was no statistically significant difference observed between mothers kept on HFD or ND during pregnancy; 66.4% vs. 58.6%; *p* = 0.104; *n* = 6/group (Figure 2B). No differences were observed in maternal daily caloric intake measured on day 20 of the pregnancy and on day 16 of the lactation, *n* = 3/group (Figure 2C). Glucose tolerance tests were performed on day 14 of the pregnancy by careful intraperitoneal injections of glucose solution (1g/kg). Fasting glucose in the HFD dams was 70.5 ± 4.93 vs. 71.8 ± 3.39 mg/dL in the ND dams (*p* = 0.83, NS). Quantification of the glycemic excursions as AUC did not reveal statistical differences (16858 ± 1248.0 in HFD groups vs. 17351 ± 1165.3 in ND groups, *p* = 0.32, NS; Figure 2D).

### 3.2. Offspring Characteristics

**Figure 3:**
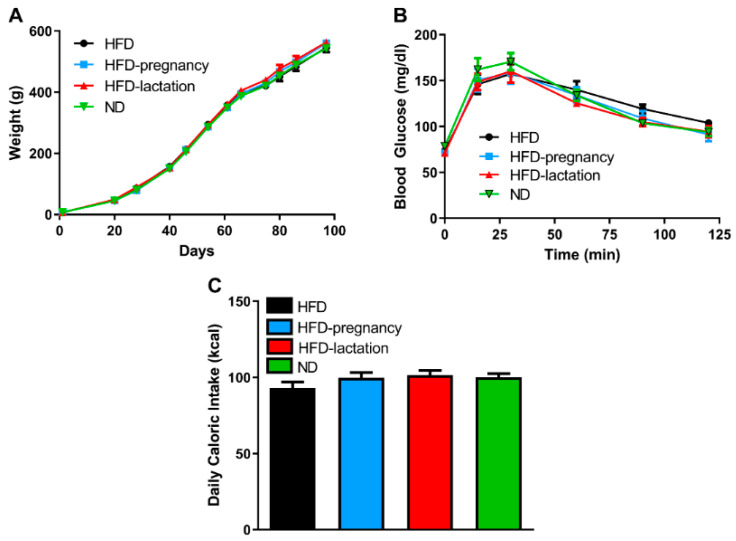
Offspring characteristics. Offspring body weight (**A**), glucose tolerance (**B**), average daily caloric intake (**C**). Group representations are the followings: black symbols and lines (**A**,**B**) or columns (**C**): HFD offspring (*n* = 9), black symbols and lines (**A**,**B**) or columns (**C**); HFD-pregnancy offspring (*n* = 9), light blue symbols and lines (**A**,**B**) or columns (**C**); HFD-lactation offspring (*n* = 7), red symbols and lines (**A**,**B**) or columns (**C**); ND group of offspring (*n* = 9), green symbols and lines (**A**,**B**) or columns (**C**). Data expressed as mean ± SEM. For more details and statistics, see Results.

Offspring body weight was recorded throughout the entire experiment. There was no difference noted in offspring body weight at birth, nor at weaning or the beginning (postnatal day 70) or end (postnatal day 90) of taste testing (Figure 3A). Glucose tolerance tests were performed on day 90–91 of life two, days following taste testing experiments, by intraperitoneal injections of glucose solution (1 g/kg) in all four groups of pups. Neither fasting glucose levels nor total area under the curve (AUC) revealed statistical differences (Figure 3B). Offspring average daily caloric intake was calculated based on daily food intake measurements during three consecutive days of each animal between 68 and 70 days of life, just prior taste testing. Daily average caloric intake was not different across offspring groups (93.0 ± 4.08, 99.85 ± 3.28, 101.56 ± 3.0, 100.0 ± 2.56 kcal in HFD, HFD-pregnancy, HFD-lactation, and ND animals, respectively; Figure 3C).

### 3.3. Brief-Access Tests

**Figure 4:**
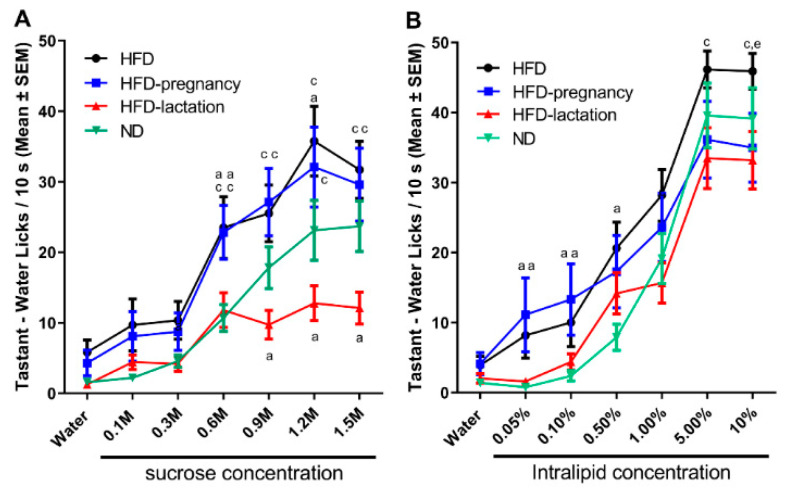
Short access taste responses to palatable sucrose and Intralipid solutions. Mean number of licks/10 s made for water and increasing concentrations of palatable solutions during taste testing at age of 10–11 weeks. Sucrose testing (**A**), and Intralipid testing (**B**). Group representations for both graphs: black lines and symbols: HFD offspring (*n* = 9); light blue lines and symbols: HFD-pregnancy offspring (*n* = 9); red lines and symbols: HFD-lactation offspring (*n* = 7); and green lines and symbols: ND group of offspring (*n* = 9). Statistical symbols represent post hoc comparisons between individual diet groups as follows: ^a^ *p* < 0.05 compared with ND; ^c^ *p* < 0.05 compared with HFD-lactation, ^e^ *p* < 0.05 comparing HFD with HFD-pregnancy. Data expressed as mean ±SEM. For more details and statistics, see Results.

Repeated measures two-way ANOVA revealed significant group (F (3, 98) = 4.411, *p* = 0.0059) and concentration (F (6, 564) = 73.83, *p* < 0.0001) effects, as well as a significant interaction (F (18, 564) = 2.641, *p* = 0.0003). Posthoc tests confirmed HFD offspring had increased 10 s lick responses for sucrose at 0.3, 0.6, and 1.2 M concentrations compared with ND pups (10.35 ± 2.70 vs. 4.76 ± 0.91; *p* = 0.042 at 0.3 M; 23.50 ± 4.38 vs. 11.11 ± 1.93; *p* = 0.013 at 0.6 M; 35.76 ± 4.96 vs. 24.05 ± 4.31; *p* < 0.05 at 1.2 M; Figure 4A). HFD-pregnancy offspring showed a similar increased response for sucrose at 0.6 M compared with ND offspring (22.81 ± 3.21 vs. 11.71 ± 4.38; *p* = 0.0101; Figure 4A). In contrast, HFD-lactation offspring showed an opposite, diminished 10 s lick response for higher concentrations of sucrose (0.9 through 1.5 M) compared with ND pups (9.73 ± 2.05 vs. 18.53 ± 2.99; *p* = 0.029 at 0.9 M; 12.78 ± 2.46 vs. 24.05 ± 4.31; *p* = 0.043 at 1.2 M; 12.12 ± 2.27 vs. 27.18 ± 3.61; *p* = 0.0094 at 1.5 M; Figure 4A).

For Intralipid, repeated measures two-way ANOVA revealed a significant main effect of concentration (F (6, 540) = 155.2, *p* < 0.0001) and interaction (F (18, 540) = 1.930, *p* = 0.0120) but no direct group effect (F (3, 90) = 2.267, *p* = 0.0860). The interaction effects, therefore, were likely driven by differences seen through the lower concentrations (<5%). In fact, posthoc tests showed HFD pups increased 10 s lick responses in the lower concentration range (0.05 through 0.5%) compared with ND offspring (8.18 ± 3.24 vs. 0.77 ± 0.35; *p* = 0.027 at 0.05%; 10.02 ± 3.45 vs. 2.37 ± 0.74; *p* = 0.035 at 0.1%; 20.62 ± 3.73 vs. 7.90 ± 1.87; *p* = 0.037 at 0.5%; Figure 4B), whereas HFD-pregnancy animals showed similar effects at 0.05 and 0.1% concentrations (11.14 ± 5.28 vs. 0.77 ± 0.35; *p* = 0.038 at 0.05%; 13.32 ± 5.09 vs. 2.37 ± 0.74; *p* = 0.025 at 0.1%; Figure 4B). Furthermore, HFD pups showed an increased 10 s lick responses for Intralipid at the highest concentration (10.0%) compared with HFD-pregnancy animals (45.88 ± 2.56 vs. 33.45 ± 4.94; *p* = 0.025; Figure 4B). Importantly, however, in contrast with the sucrose data, the HFD-lactation group was not significantly different form the ND group in the case of Intralipid testing at any of the tested concentration.

### 3.4. Two-Bottle Preference Tests

**Figure 5:**
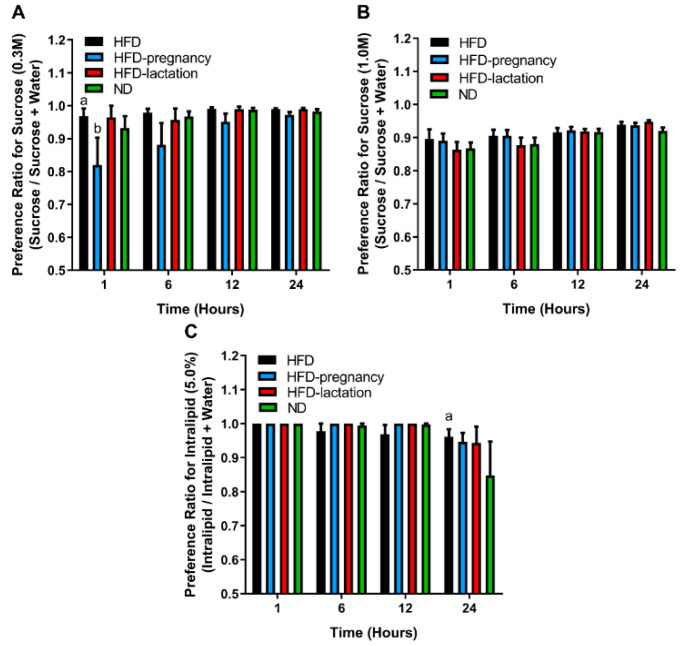
Long access two-bottle preference tests. Two-bottle preference tests for 0.3 M (**A**) and 1.0 M (**B**) sucrose and 5.0% Intralipid (**C**). Group representations are as follows for all three graphs: black columns: HFD offspring (*n* = 9); light blue columns: HFD-pregnancy offspring (*n* = 9); red columns: HFD-lactation offspring (*n* = 7); and green columns: ND group of offspring (*n* = 9). Statistical symbols (a,b) represent post hoc comparisons between groups. In the case of 0.3 M sucrose (**A**) ^a^ *p* < 0.01 compared with HFD-pregnancy; ^b^ *p* < 0.05 compared with HFD-lactation. In the case of 5.0% Intralipid (**C**) ^a^ *p* < 0.01 compared with ND. Data expressed as mean ± SEM. For more details and statistics, see Results.

To investigate the post-ingestive effects of palatable stimuli, 24 h two bottle preference tests for sucrose and Intralipid were performed following the gustometer studies, at 12 weeks of age in all four groups of offspring. Tastant + water consumptions were measured at 1, 6, 12, and 24 h time periods in each test. Preference was expressed as a ratio of tastant intake over total fluid intake (i.e., tastant plus water intake). Repeated-measures two-way ANOVA for 0.3 M sucrose (Figure 5A) revealed no significant differences for diet groups (F (3, 30) = 1.797, *p* = 0.1690) or interaction (F (9, 90) = 1.454, *p* = 0.1776), whereas there was a significant effect of time (F (3, 90) = 6.289, *p* = 0.0006). At 1 h reading of the lower sucrose concentration (0.3 M), the HFD-pregnancy group had significantly lower preferences for sucrose than the HFD (0.82 ± 0.082 vs. 0.97 ± 0.023; *p* < 0.05; Figure 5A) and HFD-lactation offspring (0.82 ± 0.082 vs. 0.96 ± 0.036; *p* < 0.05; Figure 5A). These differences, however, were not seen with longer exposure (6, 12, or 24 h). Repeated-measures two-way ANOVA for 1.0 M sucrose (Figure 5B) did not reveal any significant effects. These findings collectively suggest that post-oral effects, such as the calories consumed from the sucrose solutions, or related hormonal signals, particularly from the 1.0 M solution do not play a major role in shaping taste preferences and the observed differences in the brief-access tests.

For Intralipid solutions, repeated measures two-way ANOVA showed a significant main effect of time (F (3, 89) = 5.377, *p* = 0.0019), but no group effect (F (3, 30) = 0.5211, *p* = 0.6710) nor interaction (F (9, 89) = 0.9382, *p* = 0.4965). The time-effect was due to the 24 h reading for the HFD offspring who had a higher preference to 5.0% Intralipid solution compared with ND animals (0.96 ± 0.021 vs. 0.85 ± 0.10; *p* < 0.05; Figure 5C). This may suggest blunted post-oral feedback to Intralipid, i.e., reduced satiating effect for a fatty meal, may develop in offspring from dams that experienced prolonged HFD exposure during pregnancy and extending throughout lactation.

## 4. Discussion

The present study confirmed previous reports showing maternal HFD during pregnancy alters the sensory taste preferences in the offspring [13,22]. In addition, our study extended these observations and demonstrated a differential effect in offspring taste preferences based on timing of perinatal exposure, suggesting that pregnancy may have a greater effect over lactation on shaping sweet taste responses. Interestingly, our results showed whether the maternal HFD exposure was just during pregnancy or constant, continuing throughout lactation, the result on taste functions was similar; however, when HFD was fed exclusively during lactation, it was seemingly protective. These changes in taste preferences may, at least partially, contribute to the individual susceptibility to development of obesity and related metabolic disorders.

In the case of lactational HFD exposure, we observed a strongly blunted taste responsiveness, not graded across concentrations (Figure 4A), for sucrose but not Intralipid (Figure 4B). This type of change could be explained by defective sensory processing, when in fact the animals’ sensory taste system was not able to discriminate between different concentrations. Defective sensory processing can also lead to overeating via different mechanisms, through the activation of compensatory motivational reflexes to overcome the decreased taste sensitivity and achieve the same hedonic effect of diet by increasing propensity for more palatable, calorie dense diet during long term exposure [20,31,32]. Why the lactational HFD has an opposite directional effect and what is the real long-term impact of our findings warrants further investigation.

Meal size is controlled by orosensory stimulatory and post-ingestive inhibitory feedback (satiety signals). Thus, increased appetite and overeating can be the result of either an enhanced responsiveness to the stimulatory orosensory properties of a meal, a decreased sensitivity to post-ingestive inhibitory signals, or both [20,22,31,32]. The brief access gustometer study is designed to investigate orosensory stimulatory eating, which is highly related to taste factors, whereas the two-bottle preference test is intended to assess post-ingestive effects of palatable stimuli. We documented strong changes in taste preferences and only minimal alteration in post-ingestive feedback of tastants (Figure 4 and Figure 5), suggesting that maternal HFD exposure alters primarily and dominantly the sensory taste preferences. Although overall two-bottle studies did not reveal any major differences across the groups, two subtle differences were found which provide additional support to our observation in the short-access gustometer tests. First, the 1 h reading of the lowest sucrose concentration (0.3 M) (Figure 5A,B) suggests shifted preferences to higher concentrations due to gestational HFD exposure. Second, the 24 h reading of Intralipid two-bottle preference suggests that long term HFD exposure exaggerated potential post-ingestion regulatory deficits, likely to occur in all three HFD exposed groups, relative to the ND, resulting in increased long-term intake of 5.0% Intralipid solution (Figure 5C). Nevertheless, the present findings suggest that changes in post-ingestive feedback may not play a major role in altered taste preferences due to maternal HFD. An alternative interpretation would be that post-ingestive detection of the stimuli alters the feeding outcome triggered by oral detection only. Post-ingestive signals downstream to reward processes may also be contributory. This notion is supported further by reports showing up-regulation of expression of genes associated with reward processing [28]. In contrary, a recent study, which used a similar brief-access test, suggested that maternal HFD-induced differences in diet preferences may primarily depend on alterations in satiety signals [22]. While the reasons for this discrepancy remains unknown, it is plausible that maternal HFD exposure alters both mechanisms in a certain degree and further research using more specific methods (e.g., operant self-administration in combination with nutrient preload) is warranted to better discern contributions from oral and post-oral factors to food reward.

Obese individuals have altered taste preferences, and they need higher concentration of the palatable stimuli to achieve a similar hedonic effect [18,19,20,32]. Recent studies have shown increased sucrose preference and altered taste sensitivity overall in obese rats prior to and after development of diabetes [19,30]. Our findings occurred without obesity or impaired glycemic control, suggesting that the observed changes in taste preferences are more likely to represent a primary developmental effect on the sensory taste system, rather than secondary hemostatic changes due to obesity.

The strengths of this study are that we conducted a comprehensive approach to taste evaluation using both oral and post-oral ingestive behavior tests, as well as a design that allowed for testing of the contribution of various periods of maternal HFD exposure: gestational, lactational, or both. By this approach, we were able to distinguish between oral stimulatory and post-oral, post-ingestive feedback mechanisms, as well as separate gestational and post-gestational maternal factors related to HFD. Furthermore, our research interest was to investigate the effect of diet modification, specifically without preexisting obesity or metabolic changes caused by pregestational HFD exposure. Therefore, breeding our own animals allowed us to start the diet exposure right at the time of conception. Furthermore, the cross-fostering approach helped us clearly distinguish gestational and lactation HFD effects by eliminating the potential effect of gestational diet-induced maternal metabolic changes.

Our study has a number of limitations. For example, we had small number of dams in our experiment; thus, results of maternal characteristics should be interpreted with caution. Additionally, pups start to eat solid food around 14–15 days of life, therefore, during the last 5–6 days of the lactational period, pups were likely also eating respective maternal diet. We opted not to control for this given that early weaning has been associated with very high rates of neonatal loss and major stress.

One limitation in our design is the selective focus on sweet taste using only sucrose as a stimulus. Future studies should utilize testing responsiveness to non-nutritive sweeteners, and other taste stimuli such as salt (e.g., NaCl), bitter (e.g., quinine-HCl), and sour (e.g., citric acid) for a more comprehensive evaluation of the taste system. Further investigation is warranted to elicit the central processes of this aspect of developmental programming. Molecular genetic techniques such as qRT-PCR, microarrays, and DNA or histone methylation can be utilized to assess gene expression and epigenetic changes in the taste-related centers such as the ventral tegmental area and nucleus accumbens. Blood, milk, amniotic fluid, and placental samples of pregnant rodents would provide a very a useful approach to evaluate changes due to gestational diet modifications. Furthermore, it would be interesting to investigate whether prolonged (6–8 weeks) pregestational exposure to HFD would alter the taste preferences even further or whether ND exposure during pregnancy and lactation would able to rescue such effects.

## 5. Conclusions

Our study confirms and extends previous observations showing developmental programing of taste and food preferences in offspring due to maternal HFD. Here, we show that maternal HFD during pregnancy and lactation has markedly different effects on preferences for palatable sweet and fatty solutions in adult offspring rats and suggest that such developmental programing may primarily affect gustatory mechanisms. Future studies are warranted for determining the impact of the observed taste changes on development of obesity and metabolic disorders in a “real” food environment, i.e., with food choices available. It is important to identify specific mechanisms that may make gestational effects of maternal HFD seemingly more deleterious to development of normal taste functions, primarily for sweet taste, while maternal HFD during lactation may have an opposite, potentially protective effect.
